# Supplementary material for: High-performance bifacial perovskite solar cells enabled by single-walled carbon nanotubes
Source: Nat Commun. 2024 Mar 12;15:2245. doi: 10.1038/s41467-024-46620-1 (PMC10933432; doi:10.1038/s41467-024-46620-1)
Supplement: Supplementary file 1 — Supplementary Information [file 41467_2024_46620_MOESM1_ESM.pdf]

Supplementary Information

High-performance bifacial perovskite solar cells enabled by single-walled carbon nanotubes

Jing Zhang[†1], Xian-Gang Hu[†2,3], Kangyu Ji[4], Songru Zhao[5], Dongtao Liu[1], Bowei Li[1], Peng-Xiang Hou[2*], Chang Liu[2], Lirong Liu[5], Samuel D. Stranks[4,6], Hui-Ming Cheng[1,2,7,8*], S. Ravi P. Silva[1,9*], Wei Zhang[1,9*]

[†]These authors contributed equally: Jing Zhang, Xian-Gang Hu.

1 Advanced Technology Institute (ATI), University of Surrey, Guildford, Surrey GU2 7XH, United Kingdom.

Email: wz0003@surrey.ac.uk;cheng@imr.ac.cn;s.silva@surrey.ac.uk

2 Shenyang National Laboratory for Materials Science, Institute of Metal Research, Chinese Academy of Sciences, Shenyang, 110016, P.R.China.

Email: pxhou@imr.ac.cn

3 Advanced Interdisciplinary Research Center for Flexible Electronics, Academy of Advanced Interdisciplinary Research, Xidian University, Xi'an 710071, P.R.China

4 Cavendish Laboratory, University of Cambridge, 19 J J Thomson Avenue, Cambridge CB3 0HE, United Kingdom.

5 Centre for Environment and Sustainability, Thomas Telford (AA) building, University of Surrey, Guildford, Surrey GU2 7XH, United Kingdom.

6 Department of Chemical Engineering & Biotechnology, University of Cambridge, Philippa Fawcett Drive, Cambridge CB3 0AS, United Kingdom.

7 Faculty of Materials Science and Energy Engineering, Shenzhen University of Advanced Technology, 291 Louming Road, Shenzhen, 518107, China.

8 Shenzhen Key Lab of Energy Materials for Carbon Neutrality, Shenzhen Institute of Advanced Technology, Chinese Academy of Sciences,1068 Xueyuan Road, Shenzhen, 518055, China.

9 State Centre for International Cooperation on Designer Low-carbon & Environmental Materials (CDLCEM), School of Materials Science and Engineering, Zhengzhou University, Zhengzhou 450001, P. R. China.

* Corresponding author: Peng-Xiang Hou, email: pxhou@imr.ac.cn; Hui-Ming Cheng, email: cheng@imr.ac.cn; S. Ravi P. Silva, email: s.silva@surrey.ac.uk; Wei Zhang, email: wz0003@surrey.ac.uk.

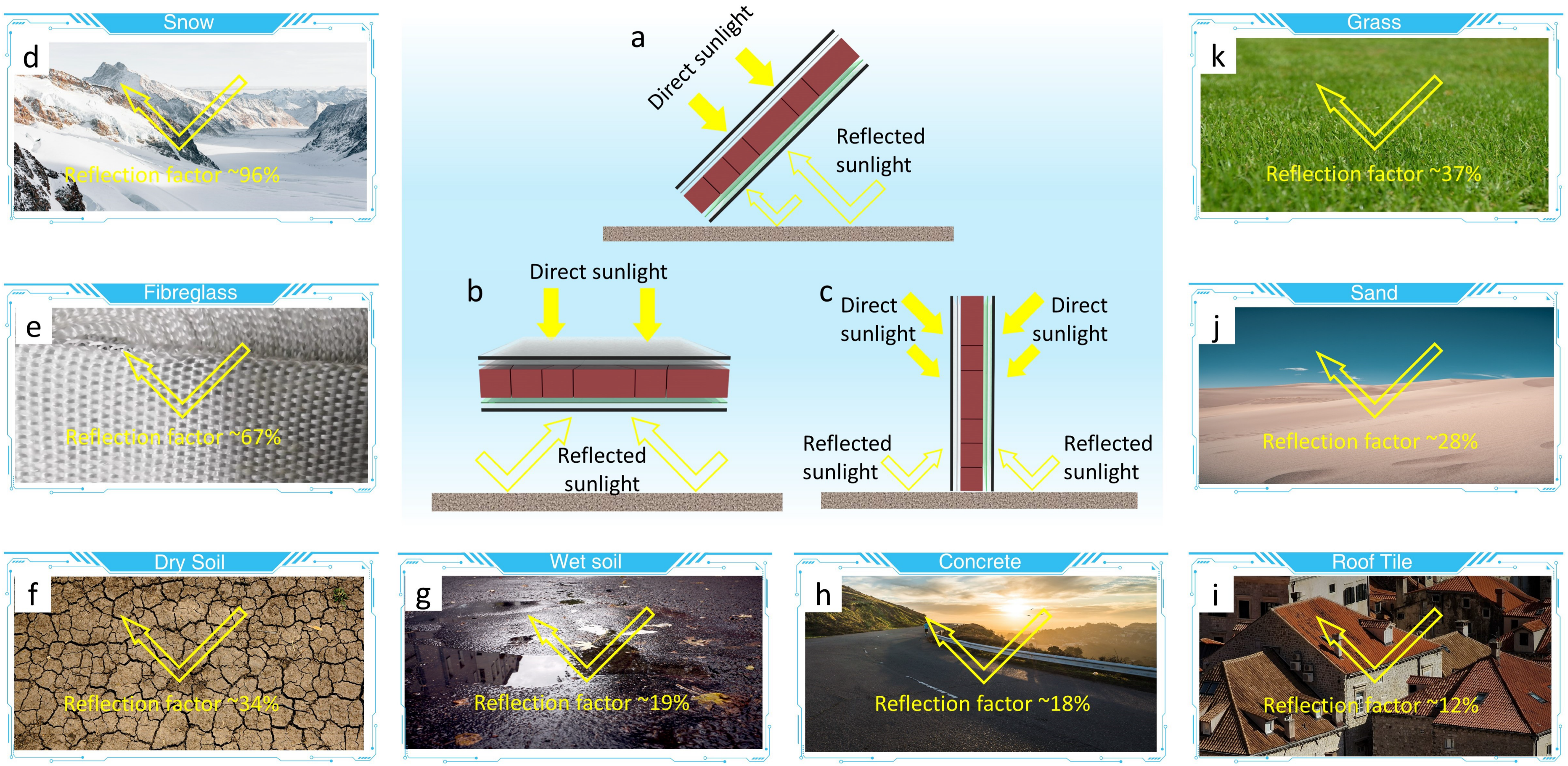

Figure S1. *The installations of bifacial PVs and several common surroundings.* **a** tilted **b** horizontal and **c** vertical installing configurations of bifacial PVs. Several common reflection surfaces and corresponding reflection factors include **d** snow(~96%, Photo by Erol Ahmed on Unsplash https://unsplash.com/photos/mountains-covered-with-snow-d3pTF3r_hwY), **e** fibreglass (~67%), **f** dry soil (~34%, Photo by Mike Erskine on Unsplash https://unsplash.com/photos/brown-and-black-concrete-floor-GKFsewk-hz0),

g wet soil (~19%, Photo by Jens Freudenau on Unsplash https://unsplash.com/photos/a-street-with-a-puddle-of-water-on-it-_9sEW7Jfd00), **h** concrete (~18%, Photo by Joshua Sortino on Unsplash https://unsplash.com/photos/person-running-on-road-street-cliff-during-golden-hour-XMcoTHgNcQA), **i** tiles (~12%, Photo by Maxim Berg on Unsplash https://unsplash.com/photos/brown-roof-tiles-during-daytime-OOEaunyf5ew), **j** sand (~28%, Photo by Colin Lloyd on Unsplash https://unsplash.com/photos/white-sand-under-blue-sky-during-daytime-Kv4bOz58rKI), and **k** grass (~37%).

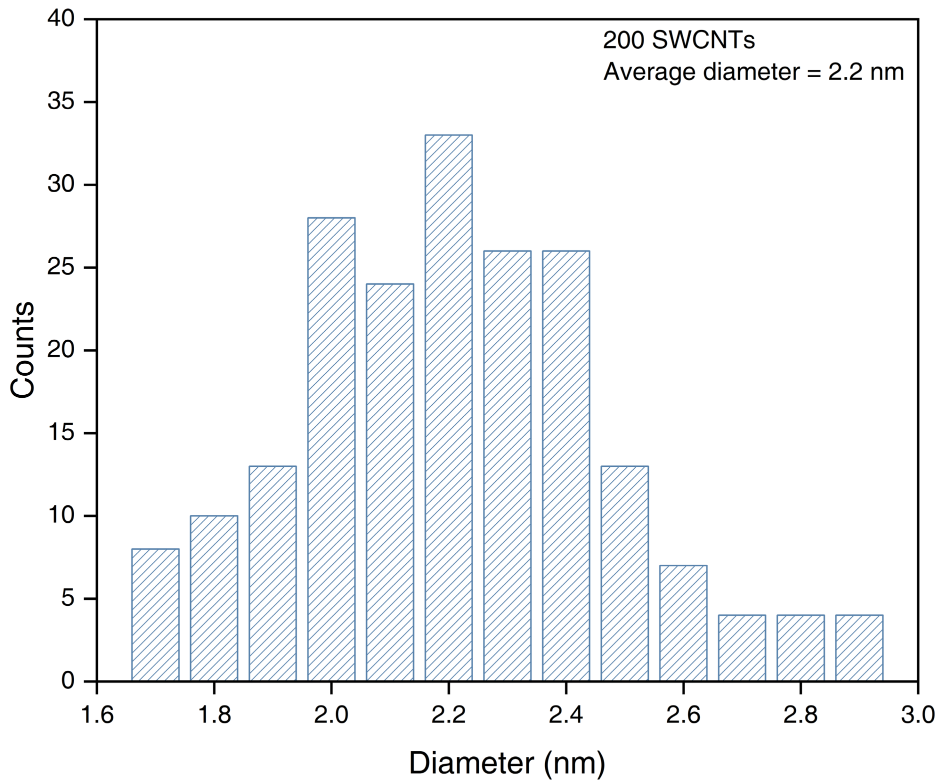

Figure S2. Diameter distribution of SWCNTs measured from TEM images of 200 nanotubes.

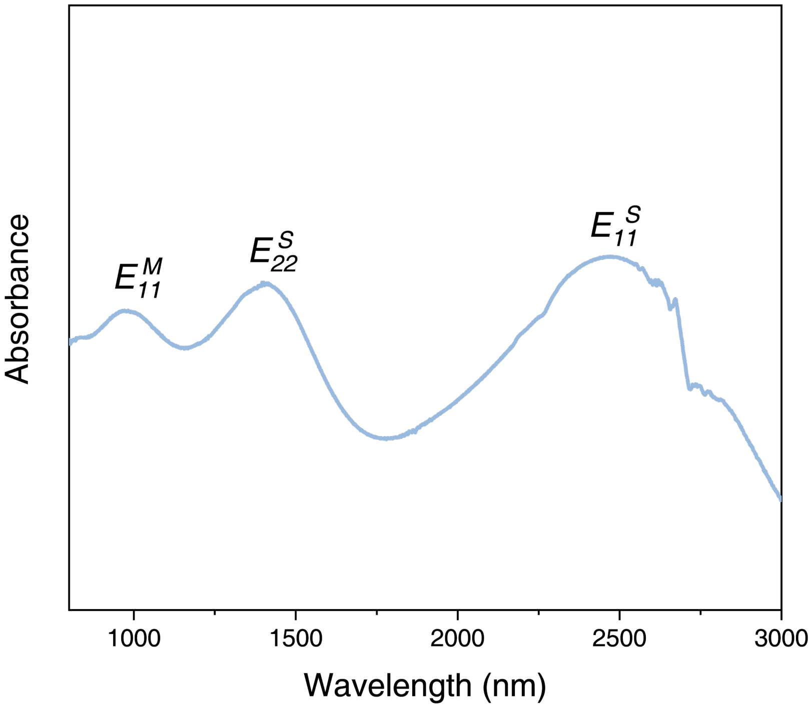

Figure S3. The optical absorbance of the pristine SWCNT film.

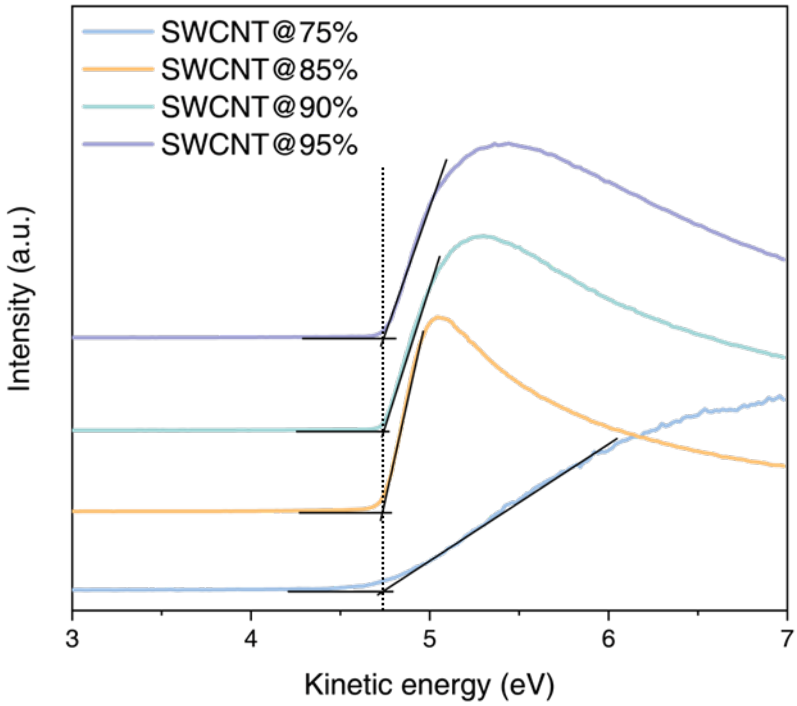

Figure S4. UPS spectra of the SWCNT network with different optical transmittances and the calculated work function is ~4.70 eV.

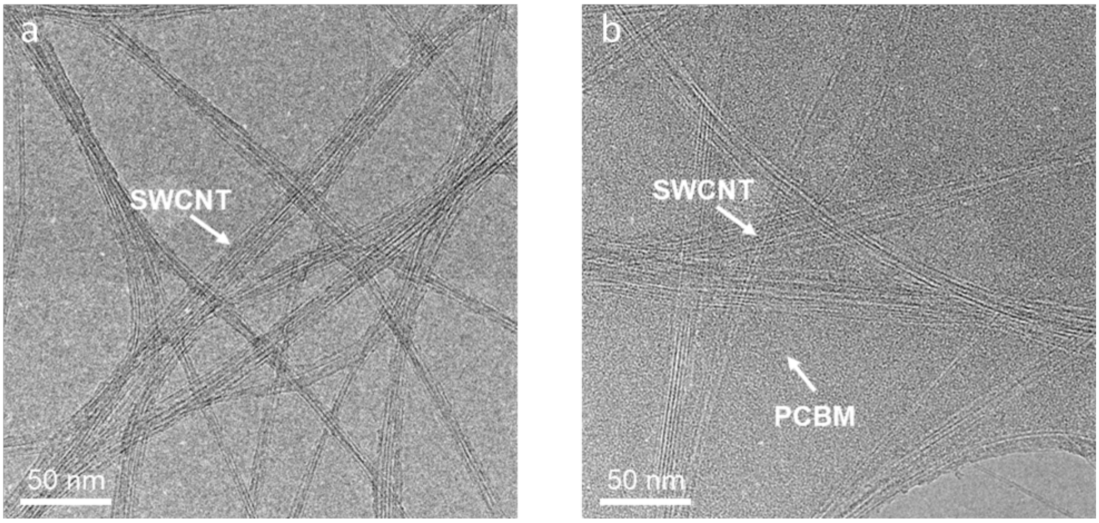

Figure S5. TEM images of a) the SWCNT film and (scale bar = 50 nm) b) the PCBM/SWCNT film (scale bar = 50 nm).

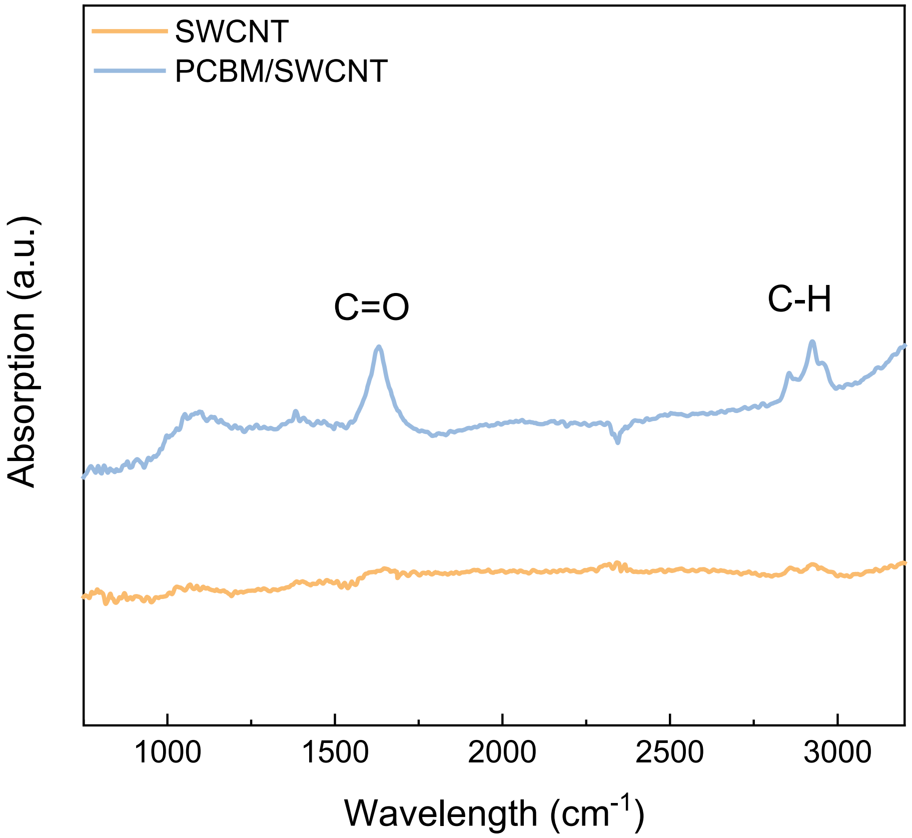

Figure S6. Fourier transform infrared spectroscopy (FTIR) transmission spectra of the SWCNT film and the PCBM/SWCNT film.

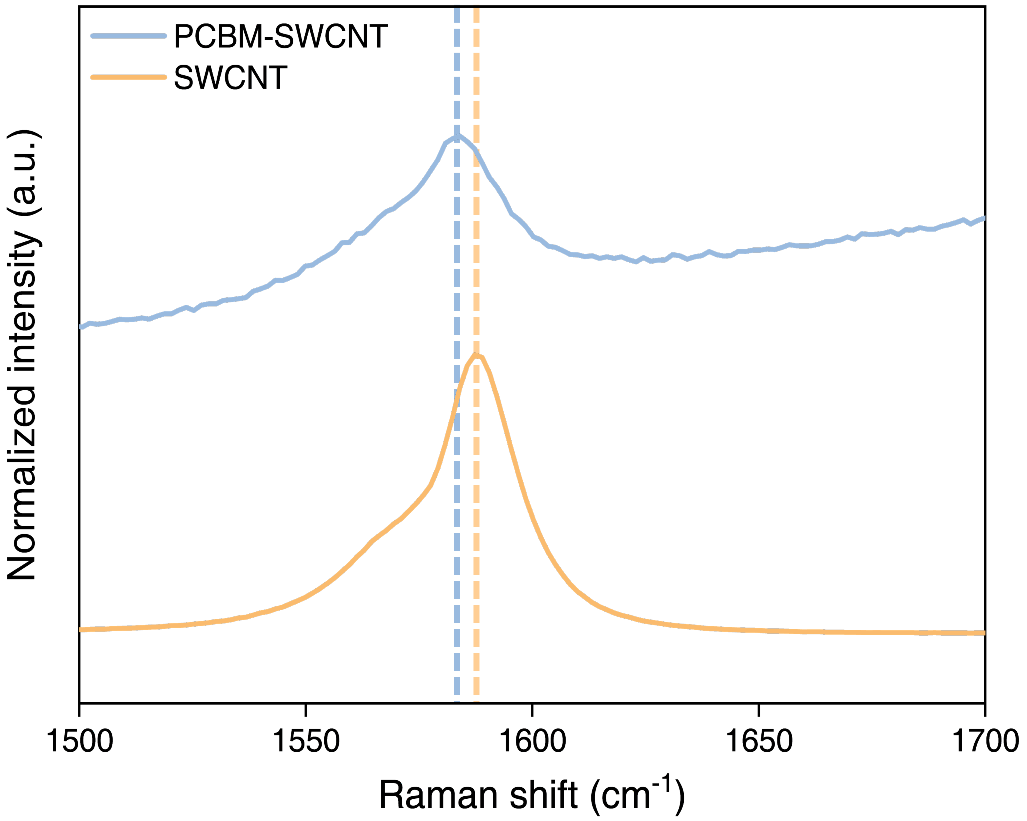

Figure S7. Raman spectra of single SWCNT and PCBM-SWCNT.

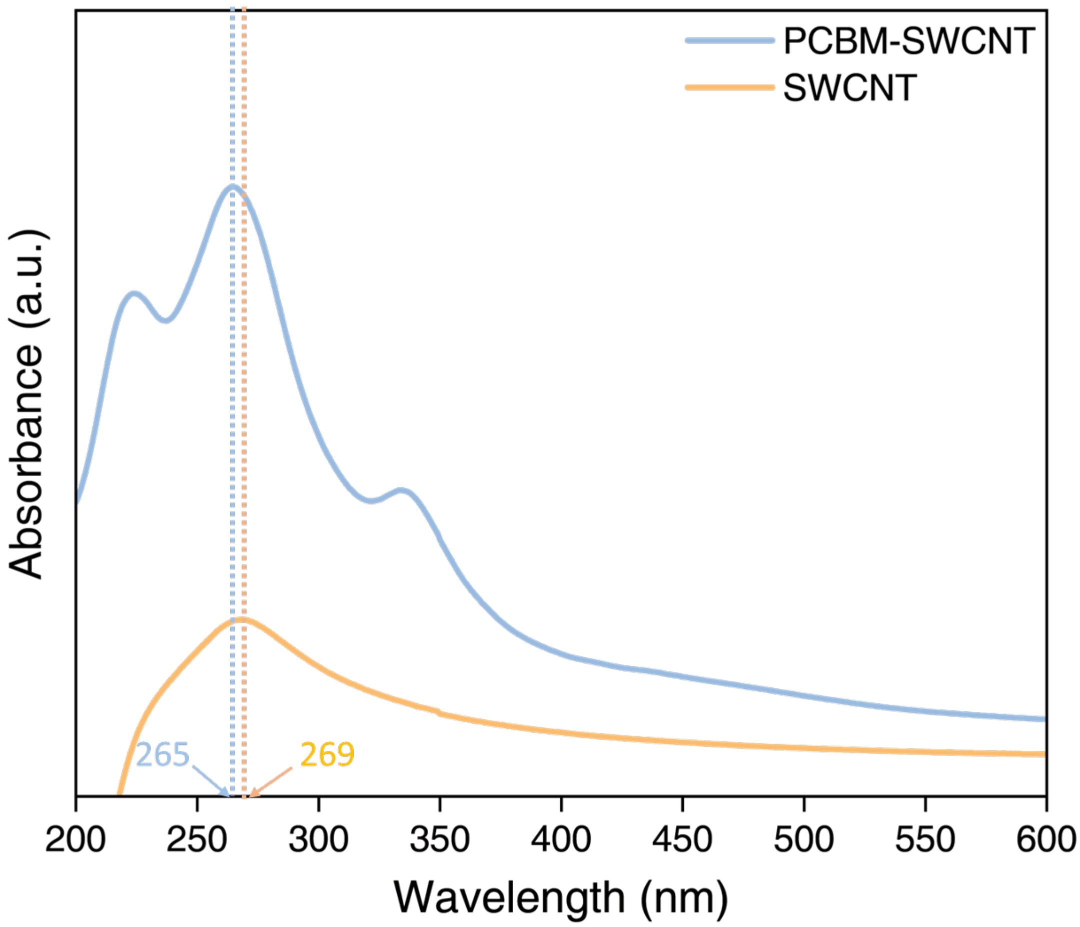

Figure S8. UV–vis spectra of SWCNT and PCBM-SWCNT.

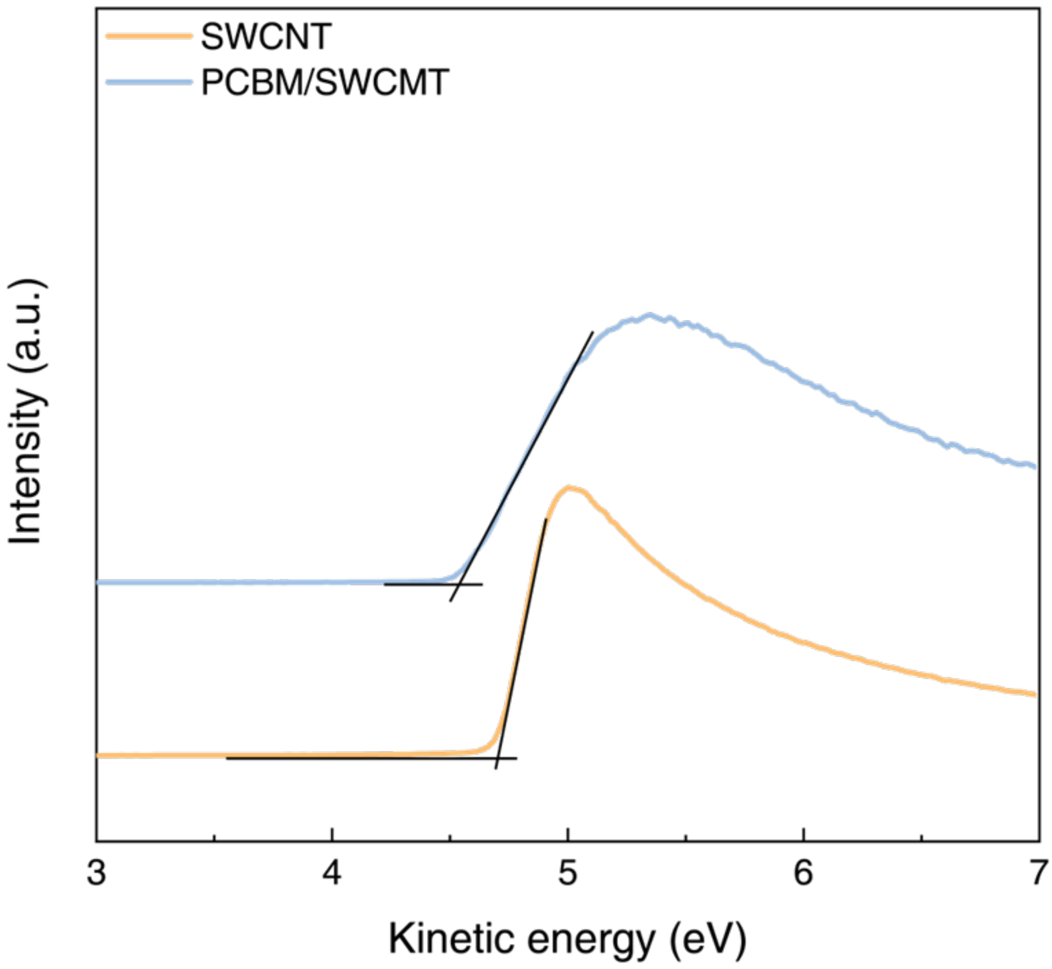

Figure S9. UPS spectra of the SWCNT film and the PCBM/SWCNT film.

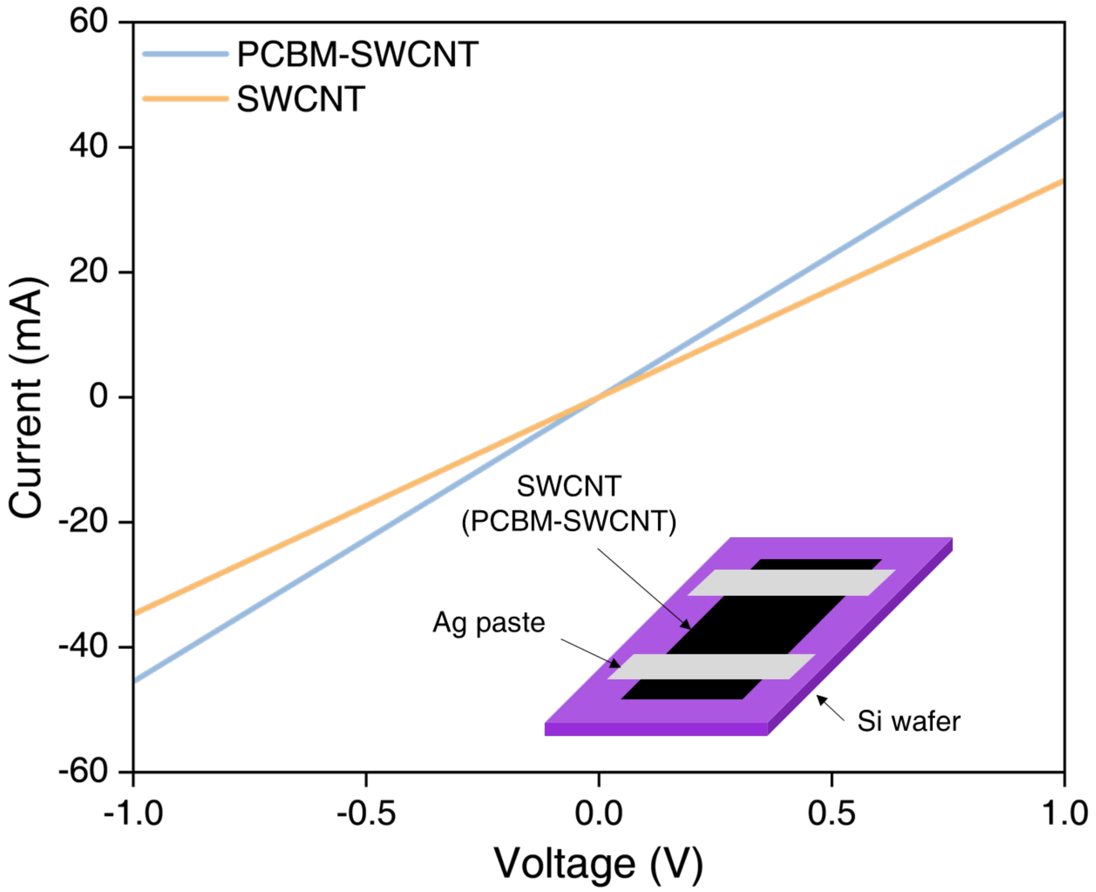

Figure S10. I-V curves of SWCNT films with and without PCBM, 28.8 Ω for SWCNT film and 21.9 Ω for SWCNT/PCBM, the inset picture shows the configuration of samples.

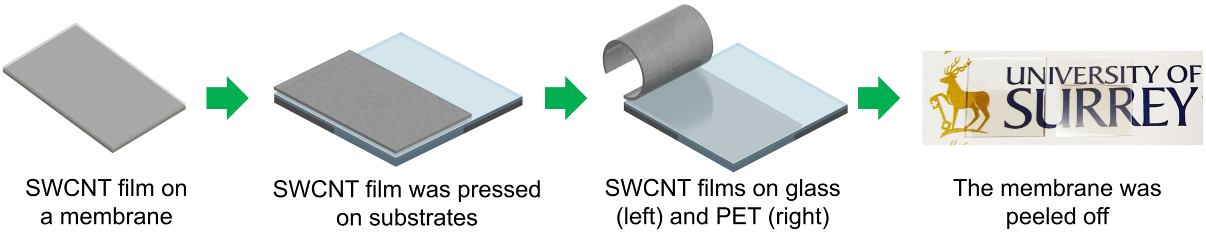

Figure S11. Dry transfer process of the SWCNT films. SWCNT films are collected on a membrane during the FCCVD process and then cut into required sizes. Press the membrane on target substrates and peel off the membrane, leaving the SWCNT films on substrates.

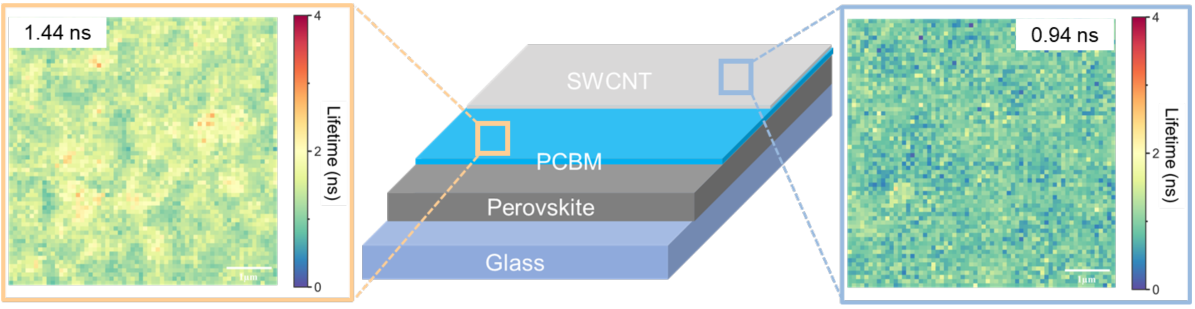

Figure S12. Half-stack device configuration and charge lifetime mapping of areas with and without SWCNT films (scale bar=1 μm).

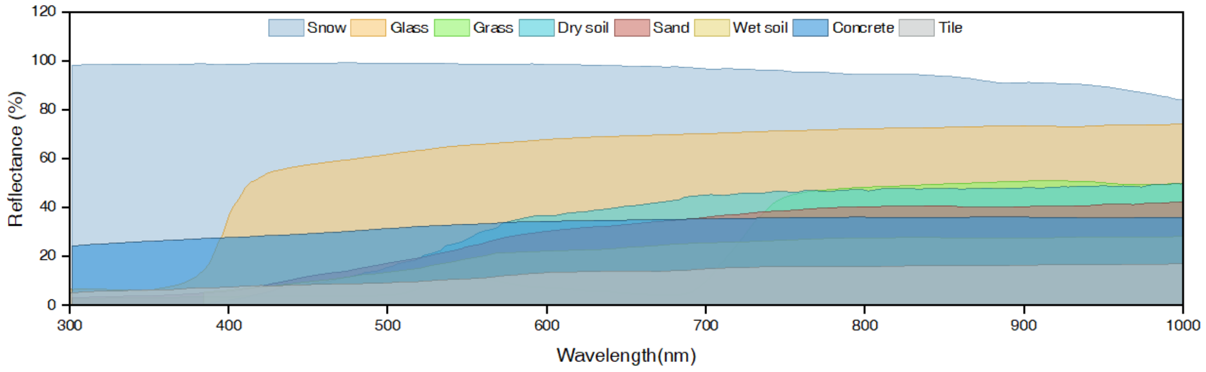

Figure S13. Reflectance spectra of some representative common ground materials. The reflectance data are replotted from data presented in NASA's ECOSTRESS Spectral Library. [1]

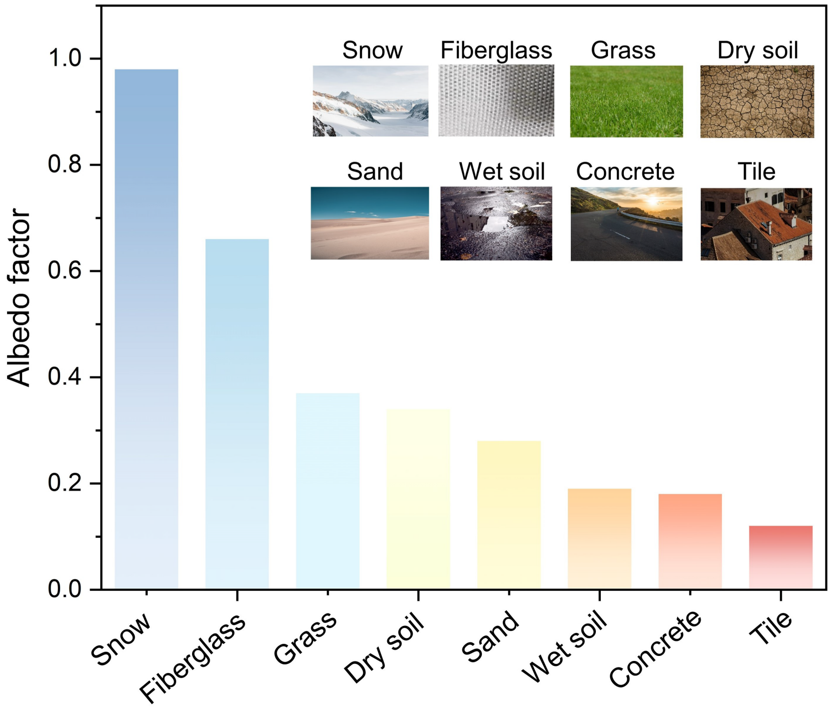

Figure S14. Albedo factor is the fraction of incoming solar radiation (short wave radiation) that is reflected by a surface. Here we estimate the albedo factors of some common ground materials in the wavelength range from 300 nm to 850 nm, which matches the perovskite absorption range.(Snow, Photo by Erol Ahmed on Unsplash https://unsplash.com/photos/mountains-covered-with-snow-d3pTF3r_hwY. Dry soil, Photo by Mike Erskine on Unsplash https://unsplash.com/photos/brown-and-black-concrete-floor-GKFsewk-hz0. Wet soil, Photo by Jens Freudenau on Unsplash https://unsplash.com/photos/a-street-with-a-puddle-of-water-on-it-_9sEW7Jfd00. Concrete, Photo by Joshua Sortino on Unsplash https://unsplash.com/photos/person-running-on-road-street-cliff-during-golden-hour-XMcoTHgNcQA. Tiles, Photo by Maxim Berg on Unsplash https://unsplash.com/photos/brown-roof-tiles-during-daytime-OOEaunyf5ew. Sand, Photo by Colin Lloyd on Unsplash https://unsplash.com/photos/white-sand-under-blue-sky-during-daytime-Kv4bOz58rKI.)The reflectance data are replotted from data presented in NASA's ECOSTRESS Spectral Library.[1]

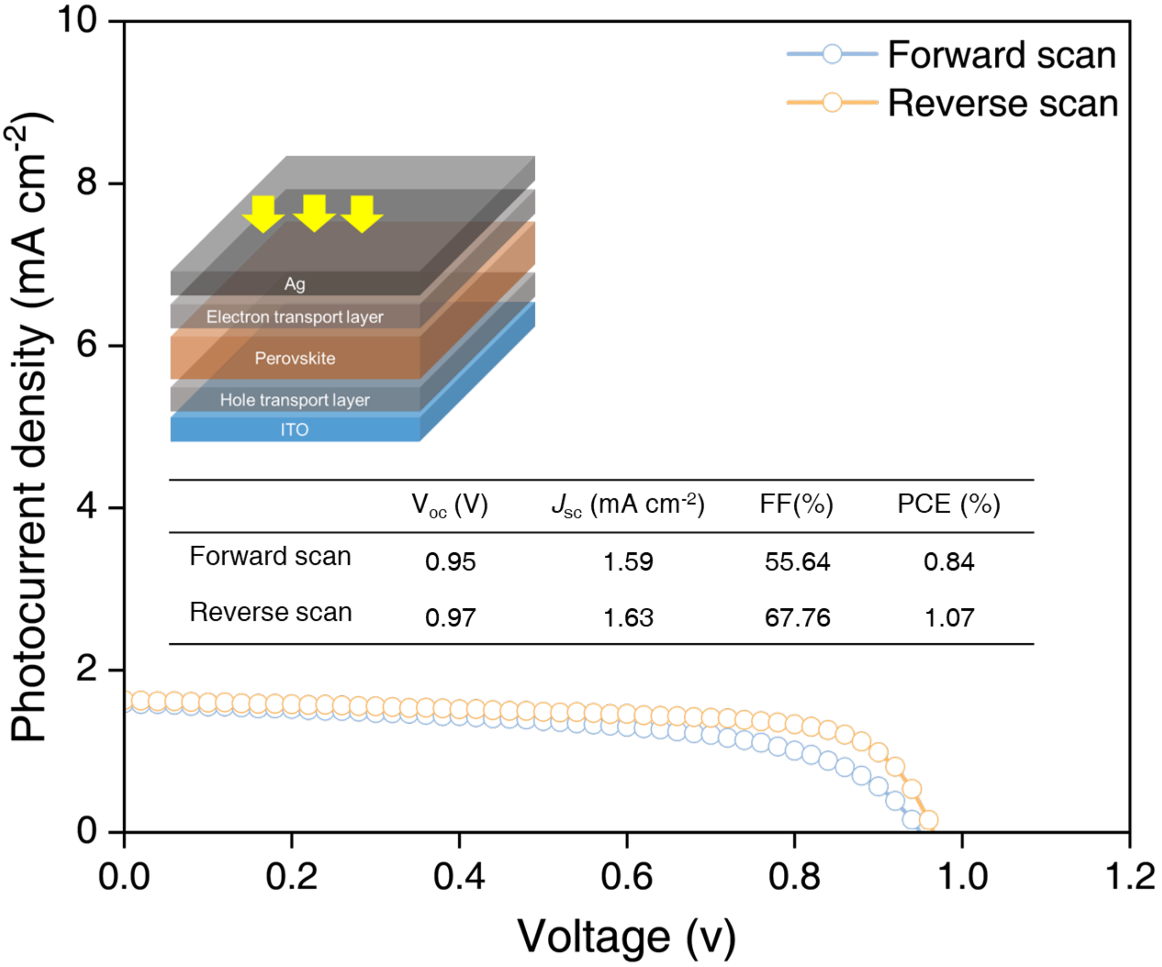

	V_{oc} (V)	J_{sc} (mA cm^{-2})	FF(%)	PCE (%)
Forward scan	0.95	1.59	55.64	0.84
Reverse scan	0.97	1.63	67.76	1.07

Figure S15. *J*-V curve of an opaque control cell with a configuration of Glass Cu:NiO_x/Perovskite/SnO2/PCBM/BCP/Ag, illuminated from the Ag side (under AM 1.5G).

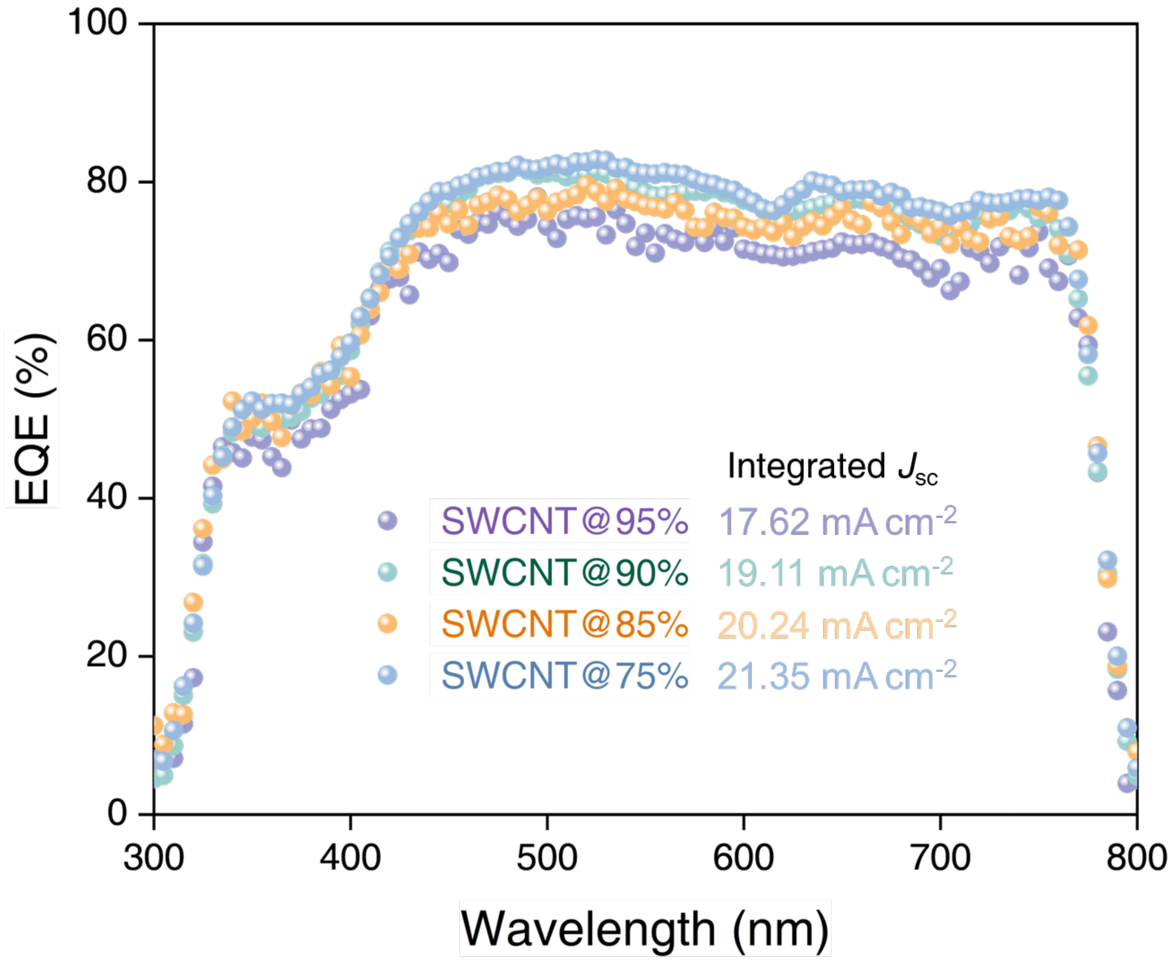

Figure S16. EQE data of all-carbon-electrode-based PSCs that have SWCNT@95%, SWCNT@90%, SWCNT@85% and SWCNT@75% as the back electrode. And the illumination is from the front side.

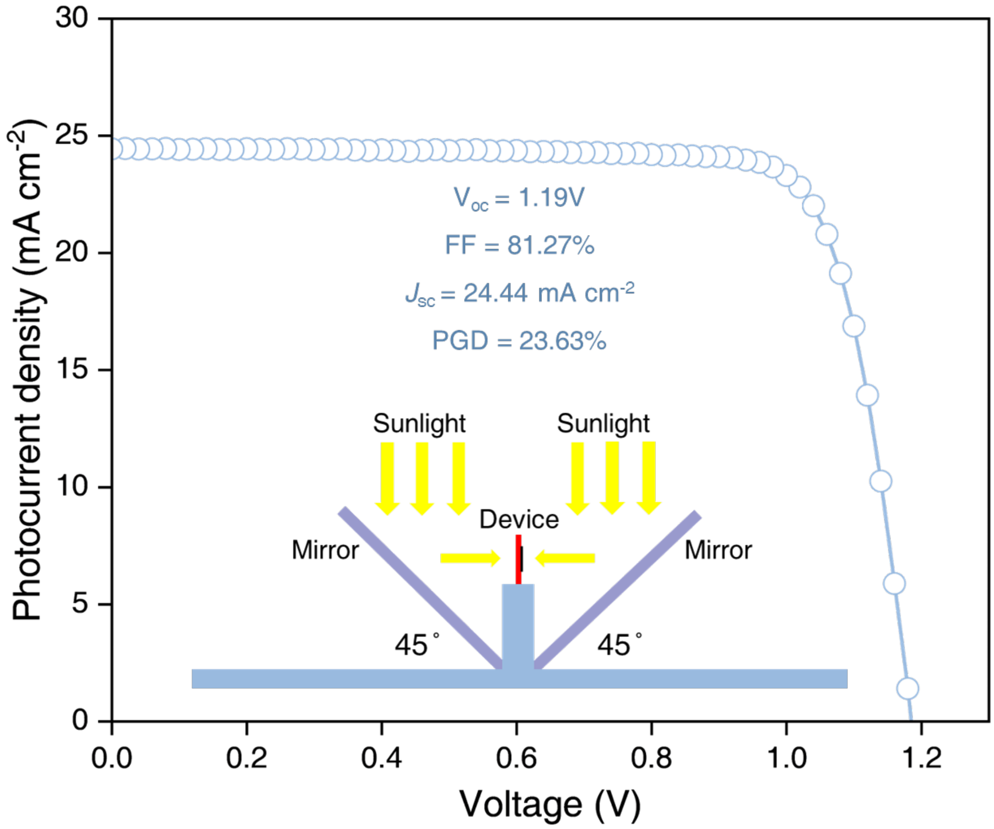

Figure S17. *J*-V curve of a control opaque device with a configuration of ITO/Cu:NiOx/Perovskite/SnO_2/PCBM/BCP/SWCNT under double light source setup.

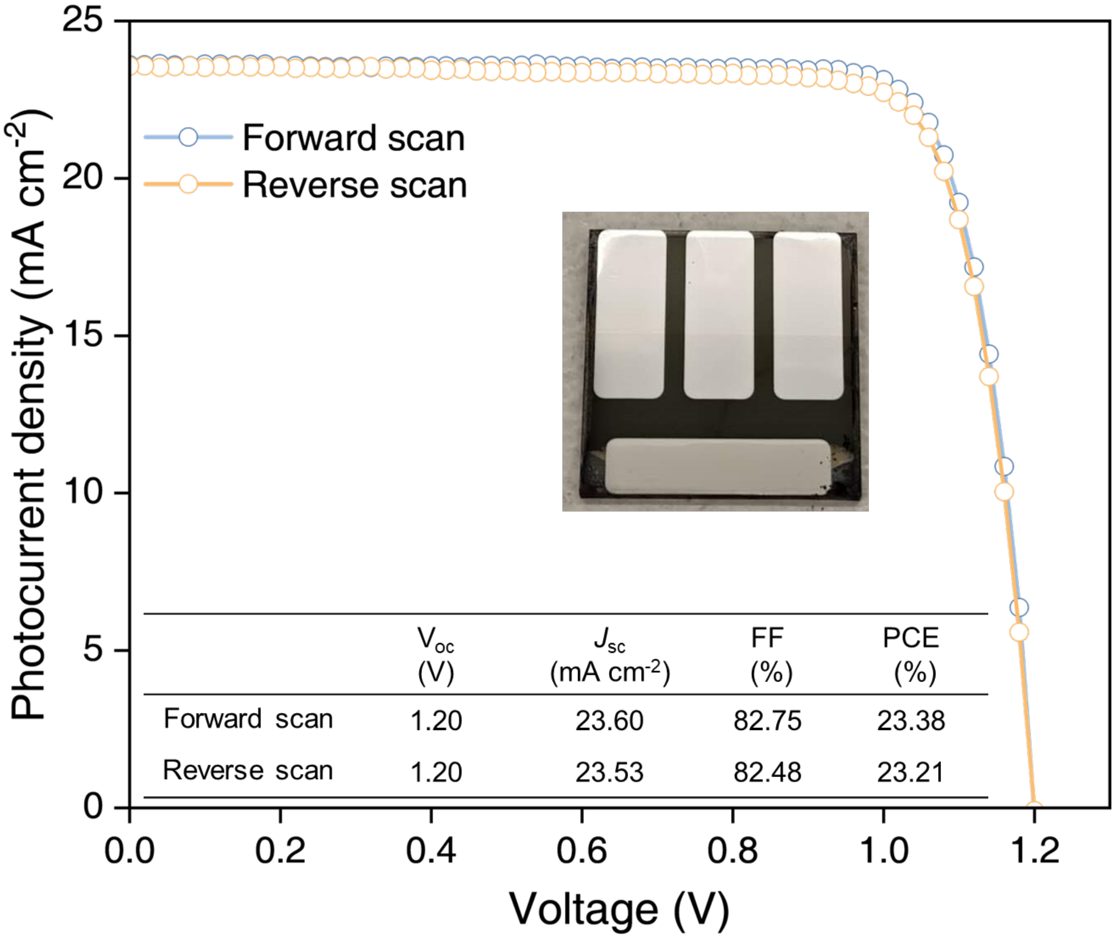

Figure S18. Photovoltaic parameters of the opaque control cell, the configuration is glass/ITO/Cu:NiO_x/Perovskite/SnO_2/PCBM/BCP/Ag.

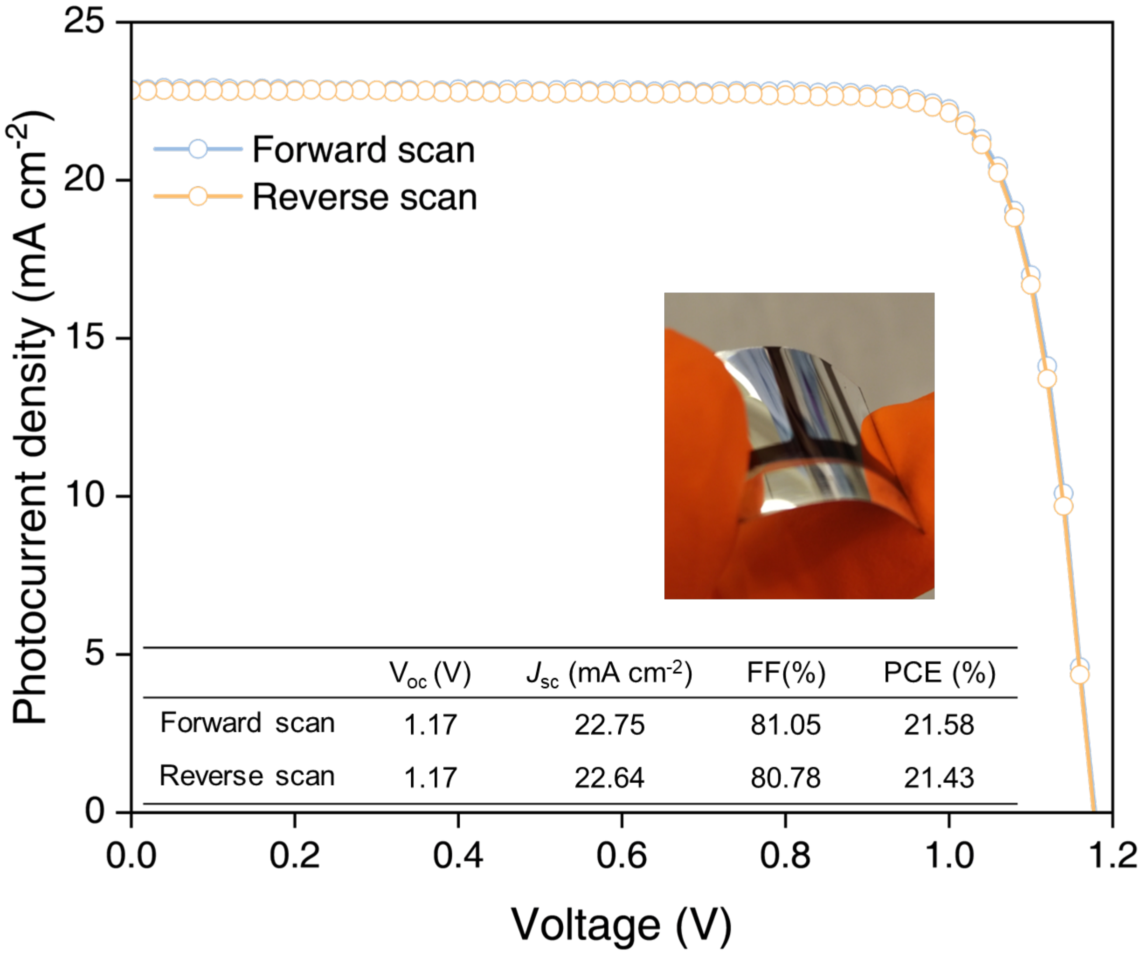

Figure S19. Photovoltaic parameters of the flexible opaque control cell, the configuration is PEN/ITO/Cu:NiO_x/Perovskite/SnO_2/PCBM/BCP/Ag.

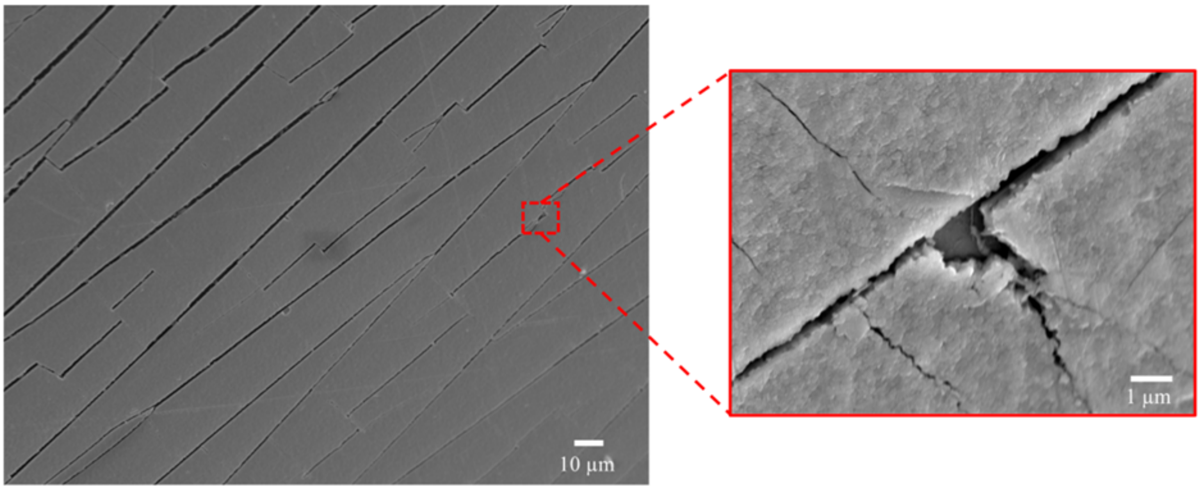

Figure S20. SEM images of cracks on ITO on PEN after being bent 100 times at a bending radius of 4mm.

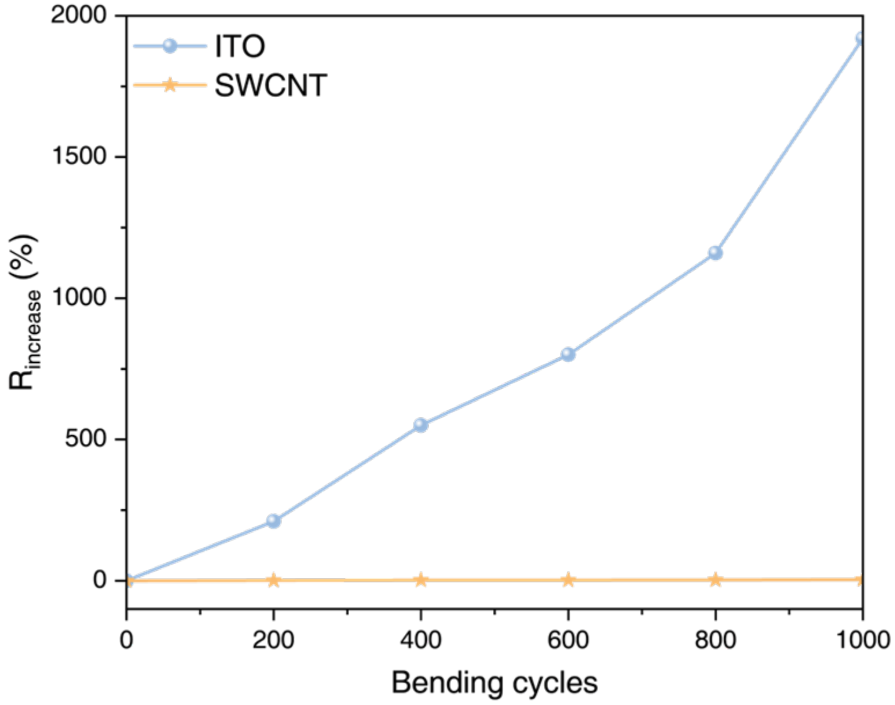

Figure S21. Variations in sheet resistance increase of the PEN-ITO and PEN-SWCNT@85% as a function of the cycles of bending.

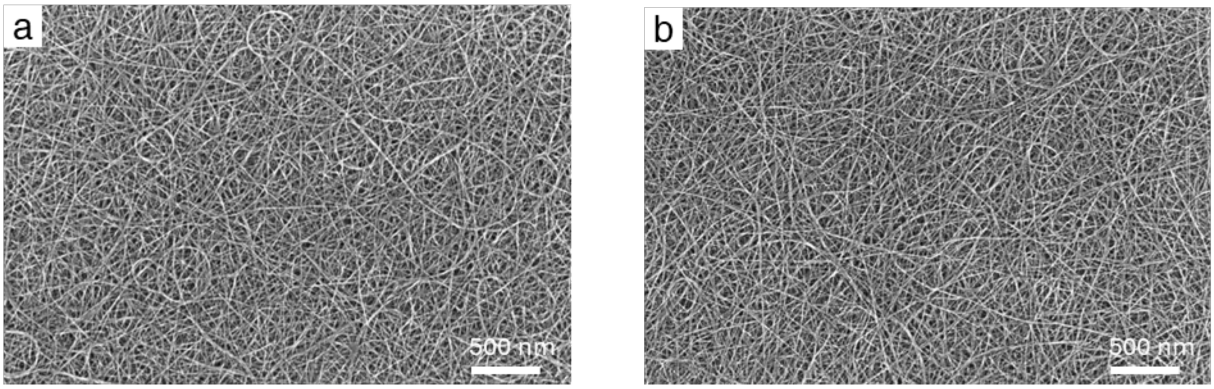

Figure S22. SEM images of SWCNT@85% **a** before and **b** after 1000 times bending.

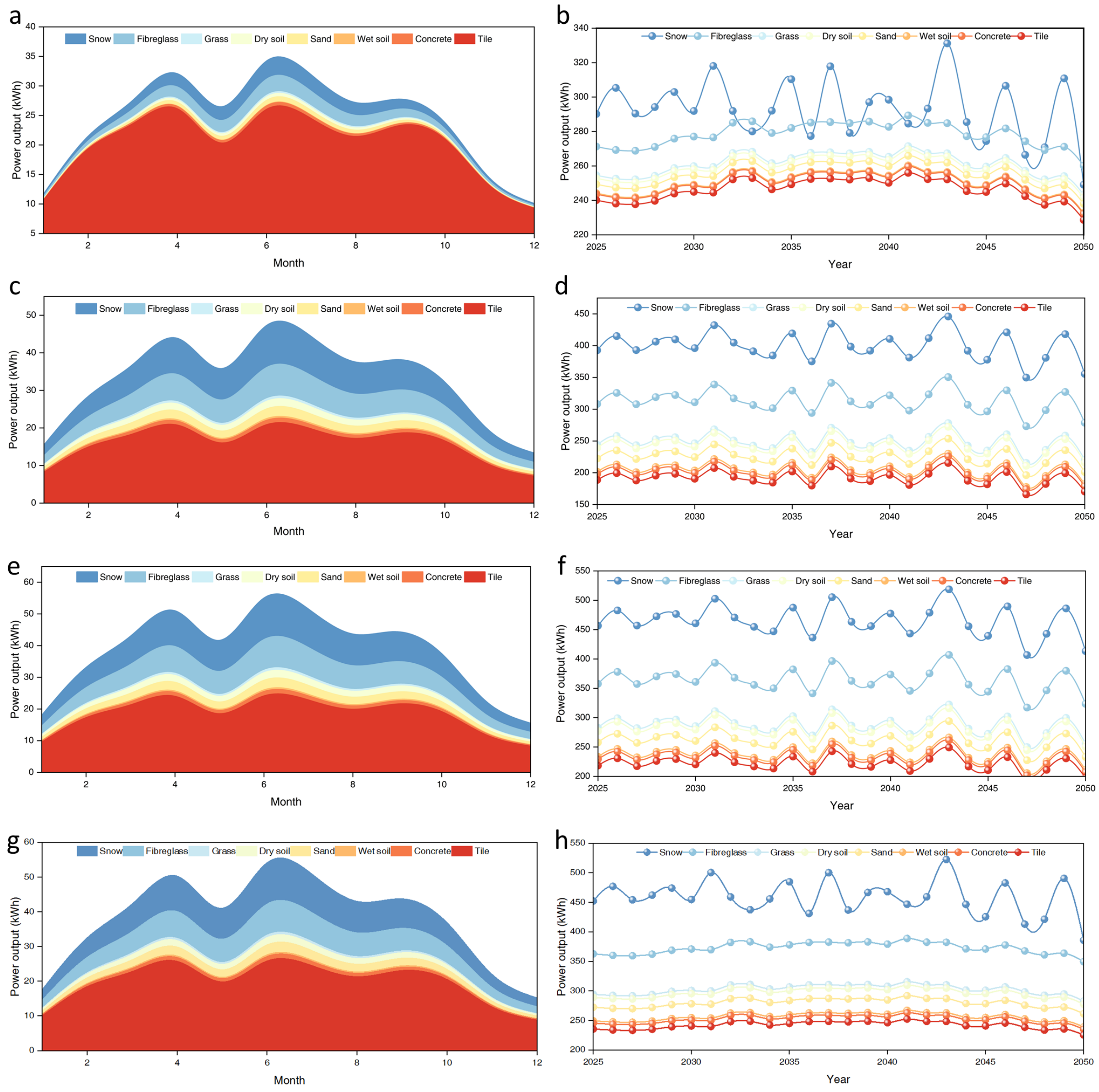

Figure S23. Power generation simulation of commercial Si solar cell for **a** one-year (2025) and **b** 26-year (from 2025 to 2050), SWCNT@95%-based PSC for **c** one-year (2025) and **d** 26-year (from 2025 to 2050), SWCNT@90%-based PSC for **e** one-year (2025) and **f** 26-year (from 2025 to 2050), and SWCNT@75%-based PSC for **g** one-year (2025) and **h** 26-year (from 2025 to 2050).

Table S1. figure of merit values of SWCNT films with different optical transmittances.

	R_{sh} (Ω sq^{-1})	T(%)	FOM(%)
SWCNT@95%	138	95	0.434
SWCNT@90%	64	90	0.545
SWCNT@85%	28	85	0.703
SWCNT@75%	17	75	0.331

Table S2. PGD summary of devices with SWCNT@95%, SWCNT@90%, SWCNT@85%, and SWCNT@75% as back electrodes under various albedo conditions.

Back SWCNT \ Condition PGD ($mW\ cm^{-2}$)	Snow	Fibreglass	Grass	Dry soil	Sand	Wet soil	Concrete	Tile
SWCNT@95%	28.66	24.11	20.39	20.11	18.99	17.59	17.31	16.75
SWCNT@90%	33.33	27.99	23.64	23.32	22.01	20.36	20.04	19.38
SWCNT@85%	36.21	30.29	25.46	25.10	23.64	21.82	21.45	20.73
SWCNT@75%	32.92	28.31	24.54	24.26	23.19	21.70	21.41	20.85

Table S3. Photovoltaic parameters of bifacial PSCs with a configuration of Glass/SWCNT@85%/Cu:NiO_x/Perovskite/SnO_2/PCBM/SWCNT (various optical transmittance), illuminated from the front and back side (15 devices were measured under each condition).

	Direction	V_{oc} (V)	J_{sc} (mA cm^{-2})	FF (%)	PCE (%)	BiFi (%)
SWCNT@95%	Front	1.08±0.03	18.22±1.55	76.28±2.31	15.07±1.21	93.36
	Back	1.07±0.04	17.21±1.62	76.37±2.50	14.01±1.15	
SWCNT@90%	Front	1.13±0.02	20.57±1.34	74.99±2.28	17.41±1.16	94.25
	Back	1.12±0.03	20.61±1.28	71.26±2.11	16.41±1.05	
SWCNT@85%	Front	1.13±0.02	20.95±1.25	78.49±2.33	18.54±1.12	98.27
	Back	1.13±0.02	20.18±1.16	79.21±2.22	18.22±1.04	
SWCNT@75%	Front	1.13±0.03	21.89±1.18	77.80±2.41	19.14±1.11	74.24
	Back	1.13±0.04	17.01±1.45	73.79±3.17	14.21±1.63	

Table S4. Summary of the state-of-art bifacial perovskite solar cells (PCE >15%, on rigid substrates) with efficiency (front and back) and bifacial factor.

No.	Front electrode (PCE)[a]	Back electrode (PCE)[b]	Bifacial factor (%)	Ref
1	FTO (16.40%)	IZO (15.26%)	93.04	[2]
2	ITO (16.07%)	Ag (8.40%)	52.27	[3]
3	ITO (20.25%)	TeO2/Ag (16.75%)	82.72	[3]
4	ITO (15.02%)	ITO (13.79%)	91.81	[4]
5	ITO (15.40%)	MoO_x/Ag/MoO_x (9.70%)	62.99	[5]
6	ITO (16.30%)	SWCNT (6.90%)	42.33	[6]
7	ITO (17.68%)	ITO (16.01%)	90.55	[7]
8	Graphene (15.10%)	Graphene (13.80%)	91.39	[8]
9	ITO (17.20%)	ITO (16.70%)	97.09	[9]
10	ITO (22.20%)	MWCNT (10.80%)	48.65	[10]
11	ITO (21.40%)	SWCNT (16.80%)	78.50	[10]
12	ITO (19.87%)	Ag (15.48%)	77.91	[11]
13	ITO (20.20%)	ITO (15.00%)	74.25	[12]
14	SWCNT (18.54%)	SWCNT (18.22%)	98.27	This work

a. PCE was measured from the front side.

b. PCE was measured from the back side.

Table S5. Photovoltaic parameters of a flexible bifacial PSC with a configuration of SWCNT@85%/Cu:NiO_x/Perovskite/SnO_2/PCBM/SWCNT@85%. (15 devices were measured under each condition)

Illumination direction	J-V direction	V_{oc} (V)	J_{sc} (mA cm^{-2})	FF (%)	PCE (%)
Front side	Forward	1.13±0.02	19.63±0.55	77.11±1.32	17.13±0.43
	Reverse	1.12±0.01	19.44±0.48	76.79±1.25	16.80±0.37
Back side	Forward	1.12±0.02	19.39±0.61	75.77±1.14	16.60±0.34
	Reverse	1.11±0.02	19.17±0.54	75.79±1.06	16.21±0.30

Table S6. Summary of the state-of-art bifacial flexible solar cells with efficiency (front and back) and bifacial factor.

Type	Front electrode (PCE)[a]	Back electrode (PCE)[b]	Bifacial factor (%)	Ref
GIGS	ITO (9.30%)	ZnMgO (2.30%)	24.73	[13]
	ITO (15.36%)	Ni-Al (6.61%)	43.03	[14]
PSC	Graphene (15.10%)	Graphene (13.80%)	91.39	[8]
	ITO (15.02%)	ITO (12.53%)	83.42	[15]
	Graphene oxide (10.11%)	ITO/Ag (9.34%)	92.38	[16]
	SWCNT (17.13%)	SWCNT (16.60)	96.91	This work

Table S7. Material cost calculation of traditional opaque PSCs (assuming the device area is 1m^2). (Prices are extracted from the Sigma-Aldrich website)

	Price	Material usage	Materials cost
Glass/ITO	\$240 per m^2	1m^2	\$240
NiO_x	\$0.2 per g	0.667g per m^2	\$0.13
Perovskite	\$1.15 per g	2.5g per m^2	\$1.05
PCBM	\$620 per g	0.026g per m^2	\$16.12
BCP/Ag	\$14 per g	0.05g per m^2	\$0.7
Total			\$258

Table S8. Material cost calculation of all-carbon-electrode-based PSCs (assuming the device area is 1m^2). (Prices are extracted from the Sigma-Aldrich website)

	Price	Material usage	Materials cost
Glass/SWCNT	\$40 per m^2	1m^2	\$40
NiO_x	\$0.2 per g	0.667g per m^2	\$0.13
Perovskite	\$1.15 per g	2.5g per m^2	\$1.05
PCBM	\$620 per g	0.026g per m^2	\$16.12
SWCNT	\$24 per m^2	1m^2	\$24
Total			\$81.3

Supplementary References

[1] S. K. Meerdink, S. J. Hook, D. A. Roberts, E. A. Abbott, Remote Sensing of Environment 2019, 230, 111196.
[2] Y.-H. Chiang, C.-C. Peng, Y.-H. Chen, Y.-L. Tung, S.-Y. Tsai, P. Chen, Journal of Physics D: Applied Physics 2018, 51, 424002.
[3] D. Chen, S. Pang, L. Zhou, X. Li, A. Su, W. Zhu, J. Chang, J. Zhang, C. Zhang, Y. Hao, Journal of Materials Chemistry A 2019, 7, 15156.
[4] T. Li, W. A. Dunlap-Shohl, D. B. Mitzi, ACS Applied Energy Materials 2020, 3, 9493.
[5] H. Liu, R. Lang, S. Jiang, W. Lu, W. Zhang, L. Feng, H. Liu, L. Wu, X. Liu, X. Wang, W. Yu, Solar Energy 2021, 228, 290.
[6] A. Elakshar, S. Tsarev, P. M. Rajanna, M. Tepliakova, L. Frolova, Y. G. Gladush, S. M. Aldoshin, P. A. Troshin, A. G. Nasibulin, ACS Applied Energy Materials 2021, 4, 13395.
[7] J. Heo, I. Jung, H. Park, J. H. Han, H. Kim, H. Park, J.-S. Park, H. Jeon, K.-T. Lee, H. J. Park, 2022, 10, 2101696.
[8] G. Jeong, D. Koo, J.-H. Woo, Y. Choi, E. Son, F. Huang, J.-Y. Kim, H. Park, ACS Applied Materials & Interfaces 2022, 14, 33297.
[9] M. Najafi, M. Theelen, H. Fledderus, D. Zhang, V. Zardetto, B. van Aken, S. Veenstra, 2022, 6, 2100621.
[10] C. Zhang, M. Chen, F. Fu, H. Zhu, T. Feurer, W. Tian, C. Zhu, K. Zhou, S. Jin, S. M. Zakeeruddin, A. N. Tiwari, N. P. Padture, M. Grätzel, Y. Shi, Energy & Environmental Science 2022, 15, 1536.
[11] L. Fan, W. Lü, W. Hu, D. Han, S. Yang, D. Wang, Z. Mai, F. Wang, H. Liu, J. Yang, L. Yang, Materials Chemistry Frontiers 2022, 6, 2061.
[12] H. Gu, C. Fei, G. Yang, B. Chen, M. A. Uddin, H. Zhang, Z. Ni, H. Jiao, W. Xu, Z. Yan, J. Huang, Nature Energy 2023.
[13] A. Mavlonov, T. Nishimura, J. Chantana, Y. Kawano, T. Masuda, T. Minemoto, Solar Energy 2020, 211, 1311.
[14] S.-C. Yang, T.-Y. Lin, M. Ochoa, H. Lai, R. Kothandaraman, F. Fu, A. N. Tiwari, R. Carron, Nature Energy 2023, 8, 40.
[15] S. Li, C. Wang, D. Zhao, Y. An, Y. Zhao, X. Zhao, X. Li, Nano Energy 2020, 78, 105378.
[16] M.-A. Park, S. J. Sung, Y. J. Ahn, I. Hong, I. J. Park, C. R. Park, J. Y. Kim, ACS Applied Energy Materials 2021, 4, 8824.
