# Supplementary material for: High-performance bifacial perovskite solar cells enabled by single-walled carbon nanotubes
Source: Nat Commun. 2024 Mar 12;15:2245. doi: 10.1038/s41467-024-46620-1 (PMC10933432; doi:10.1038/s41467-024-46620-1)
Supplement: Supplementary file 3 — Reporting Summary [file 41467_2024_46620_MOESM3_ESM.pdf]

Corresponding author(s): Pengxiang Hou, Hui-Ming Cheng, S. Ravi P. Silva, Wei Zhang

☐ Initial submission ☐ Revised version ☒ Final submission

Solar Cells Reporting Summary

Nature Research wishes to improve the reproducibility of the work that we publish. This form is intended for publication with all accepted papers reporting the characterization of photovoltaic devices and provides structure for consistency and transparency in reporting. Some list items might not apply to an individual manuscript, but all fields must be completed for clarity.

For further information on Nature Research policies, including our data availability policy, see Authors & Referees.

Experimental design

Please check: are the following details reported in the manuscript?

1. Dimensions

Item	Answer	Details
Area of the tested solar cells	☒ Yes ☐ No	See *Methods* section.
Method used to determine the device area	☒ Yes ☐ No	See *Methods* section.

2. Current-voltage characterization

Item	Answer	Details
Current density-voltage (J-V) plots in both forward and backward direction	☒ Yes ☐ No	Figure S15, Figure S18, Figure S19, Table S3 and Table S5.
Voltage scan conditions<br>*For instance: scan direction, speed, dwell times*	☒ Yes ☐ No	See *Methods* section.
Test environment<br>*For instance: characterization temperature, in air or in glove box*	☒ Yes ☐ No	See *Methods* section.
Protocol for preconditioning of the device before its characterization	☐ Yes ☒ No	No preconditioning protocol.
Stability of the J-V characteristic<br>*Verified with time evolution of the maximum power point or with the photocurrent at maximum power point; see ref. 7 for details.*	☒ Yes ☐ No	See Figure 3e.

3. Hysteresis or any other unusual behaviour

Item	Answer	Details
Description of the unusual behaviour observed during the characterization	☐ Yes ☒ No	No hysteresis or other unusual behaviour was observed.
Related experimental data	☐ Yes ☒ No	No hysteresis or other unusual behaviour was observed.

4. Efficiency

Item	Answer	Details
External quantum efficiency (EQE) or incident photons to current efficiency (IPCE)	☒ Yes ☐ No	See Figure S16.
A comparison between the integrated response under the standard reference spectrum and the response measure under the simulator	☒ Yes ☐ No	There are less than 5% errors between the integrated response under the standard reference spectrum and the response measure under the simulator.
For tandem solar cells, the bias illumination and bias voltage used for each subcell	☐ Yes ☒ No	We only fabricated single junction devices.

5. Calibration

Item	Answer	Details
Light source and reference cell or sensor used for the characterization	☒ Yes ☐ No	See *Methods* section.

Confirmation that the reference cell was calibrated and certified	[x] Yes	See *Methods* section.
	[ ] No	
Calculation of spectral mismatch between the reference cell and the devices under test	[ ] Yes	
	[x] No	

6. Mask/aperture

Size of the mask/aperture used during testing	[x] Yes	See *Methods* section.
	[ ] No	
Variation of the measured short-circuit current density with the mask/aperture area	[ ] Yes	
	[x] No	The aperture area is fixed for all the devices.

7. Performance certification

Identity of the independent certification laboratory that confirmed the photovoltaic performance	[ ] Yes	
	[x] No	Devices are not certified.
A copy of any certificate(s) *Provide in Supplementary Information*	[ ] Yes	
	[x] No	

8. Statistics

Number of solar cells tested	[x] Yes	Table S3, Table S5.
	[ ] No	
Statistical analysis of the device performance	[ ] Yes	
	[x] No	

9. Long-term stability analysis

Type of analysis, bias conditions and environmental conditions *For instance: illumination type, temperature, atmosphere humidity, encapsulation method, preconditioning temperature*	[x] Yes	Figure 3c, Figure 3d, Figure 3e, Figure 3c , Figure 4c and *Methods* section.
	[ ] No	

Further reading

1. Shrotriya, V. *et al*. Accurate measurement and characterization of organic solar cells. *Adv. Funct. Mater.* **16**, 2016–2023 (2006).
2. Dennler, G. *et al*. The value of values. *Mat. Today* **10**, 56 (2007).
3. Cravino, A., Schilinsky, P. & Brabec, C. J. Characterization of organic solar cells: the importance of device layout. *Adv. Funct. Mater.* **17**, 3906–3910 (2007).
4. Reese, M. O. *et al*. Consensus stability testing protocols for organic photovoltaic materials and devices. *Sol. Energ. Mat. Sol. C* **95**, 1253–1267 (2011).
5. Snaith H. .J. The perils of solar cell efficiency measurements. *Nat. Photon*. **6**, 337–340 (2012).
6. Luber, E. J. & Buriak, J. M. Reporting performance in organic photovoltaic devices. *ACS Nano* **7**, 4708–4714 (2013).
7. Snaith, H. J. *et al*. Anomalous hysteresis in perovskite solar cells. *J. Phys. Chem. Lett*. **5**, 1511–1515 (2014).
8. Grätzel M. The light and shade of perovskite solar cells. *Nat. Mat*. **13**, 838–842 (2014).
9. Zimmermann E. *et al*. Erroneous efficiency reports harm organic solar cell research. *Nat. Photon*. **8**, 669–672 (2014).
10. Beard M.C., Luther J.M. & Nozik A.J. The promise and challenge of nanostructured solar cells. *Nat. Nanotech*. **9**, 951–954 (2014).
11. Timmreck, R. *et al*. Characterization of tandem organic solar cells. *Nat. Photon*. **9**, 478–479 (2015).

A number of international committees develop industry standards on the characterization of photovoltaic technologies (for example ASTM-E44 and IEC-TC 82), which can provide guidance for academic research.

This checklist template is licensed under a Creative Commons Attribution 4.0 International License, which permits use, sharing, adaptation, distribution and reproduction in any medium or format, as long as you give appropriate credit to the original author(s) and the source, provide a link to the Creative Commons license, and indicate if changes were made. The images or other third party material in this article are included in the article's Creative Commons license, unless indicated otherwise in a credit line to the material. If material is not included in the article's Creative Commons license and your intended use is not permitted by statutory regulation or exceeds the permitted use, you will need to obtain permission directly from the copyright holder. To view a copy of this license, visit http://creativecommons.org/licenses/by/4.0/
